# Global Communication Practices and Their Impact on Patient Caregivers’ Satisfaction in the Surgical Waiting Area: A Scoping Review

**DOI:** 10.3390/healthcare13121408

**Published:** 2025-06-12

**Authors:** Dnyata Dhanajirao Pandit, Sai Bhavana, Anitha Nileshwar, Latha T, Kirthinath Ballala, Elstin Anbu Raj, Somu G, Rajesh Kamath

**Affiliations:** 1 Department of Hospital Administration, Kasturba Medical College, Manipal Academy of Higher Education, Manipal 576104, India; dnyata.kmcmpl2023@learner.manipal.edu; 2 Department of Healthcare and Hospital Management, Prasanna School of Public Health, Manipal Academy of Higher Education, Manipal 576104, India; bhavana.psphmpl2023@learner.manipal.edu; 3 Department of Anesthesiology, Kasturba Medical College, Manipal Academy of Higher Education, Manipal 576104, India; anitha.rshenoy@manipal.edu; 4 College of Nursing, AIIMS Bibinagar, Bibinagar 508126, India; latha.con@aiimsbibinagar.edu.in; 5 Department of Community Medicine, Kasturba Medical College, Manipal Academy of Higher Education, Manipal 576104, India; kirthinath.ballala@manipal.edu; 6 Centre for Evidence-informed Decision Making, Prasanna School of Public Health, Manipal Academy of Higher Education, Manipal 576104, India; s.elstin@manipal.edu

**Keywords:** healthcare communication, patient caregiver satisfaction, global communication practices, surgical settings, digital communication practices, caregiver experience in hospitals, hospital communication strategies, scoping review

## Abstract

**Background/Objectives:** Effective communication between healthcare professionals and patient caregivers is paramount in the surgical waiting area, particularly during periods of heightened stress and emotional vulnerability. Globally, communication practices exhibit considerable variability, from traditional face-to-face interactions to integrating advanced digital technologies. Despite innovations, a comprehensive understanding of the impact of diverse communication strategies on patient caregiver satisfaction remains underdeveloped. This scoping review was designed to systematically map the existing literature on healthcare communication practices and identify strategies that may influence satisfaction among patient caregivers. **Methods:** A thorough search of multiple databases—Scopus, PubMed, CINAHL, Embase, ProQuest, Web of Science, the Cochrane Library, and clinical trial registries—was conducted. Only studies published in English or those for which an English full text was accessible were included. Eligible studies were those undertaken in hospital settings, including operating theaters, surgical units, surgical waiting areas, postoperative intensive care units, emergency departments, and other clinical areas focusing on patient caregivers. The review adhered to the methodological framework recommended by the Joanna Briggs Institute for scoping reviews and was reported following the most recent PRISMA-ScR guidelines. **Results:** Ultimately, five studies met the inclusion criteria. The selection process involved a structured search utilizing Medical Subject Headings (MeSH), keywords, and index terms, supplemented by manual reference list screening. Initial screening was performed based on titles and abstracts, followed by full-text evaluation using a standardized selection form. Data extraction focused on the communication methods, study designs, and outcomes related to patient caregiver satisfaction. The findings are synthesized narratively and presented through tables and figures, offering a comprehensive overview of global communication practices and their influence on patient caregiver satisfaction in surgical environments. Across the five included studies, digital communication interventions such as SMS, mobile apps, or video updates reported improved patient caregiver satisfaction (e.g., 70.8% in Canada and 97% in Switzerland) and also reduced patient caregiver anxiety (e.g., STAI score ≥ 44) in 74.2% of Ethiopian patient caregivers. Worldwide evidence highlights the practical importance of tailored digital communication practices to support providing timely and accessible information to patient caregivers, while also revealing gaps linked to insurance status, digital literacy, and various communication approaches in healthcare systems. **Conclusions:** The five studies included showed considerable variation in communication practices across surgical settings. The main findings reveal that structured, timely, and transparent communication, mainly via digital tools such as SMS updates and mobile applications, enhanced patient caregiver satisfaction and alleviated their emotional distress too. Nevertheless, gaps were identified in postoperative communication, and challenges, such as technological accessibility, digital literacy skills of patient caregivers, and inconsistent methods for measuring satisfaction outcomes, were noted across studies. This scoping review identified the different types of healthcare communication practices adopted globally in surgical care settings and also demonstrated their influence on patient caregiver satisfaction. Traditional and digital communication practices both have their significant impact on patient caregiver experiences in surgical healthcare settings, focusing more on timely and consistent real-time updates and culturally sensitive information. Addressing the existing communication gaps and having tailored communication approaches to specific contexts may lead to improved patient caregiver support and surgical outcomes.

## 1. Introduction

Communication between healthcare providers and patient caregivers is essential for ensuring quality care and promoting positive patient and patient caregiver experiences, especially in stressful environments such as surgical waiting areas. Patient caregivers often experience significant uncertainty and stress while waiting for updates during procedures, making effective communication a critical factor in reducing emotional turmoil and enhancing satisfaction [1,2]. Despite its importance, there is notable variability in how communication is delivered globally. Different healthcare systems employ various strategies depending on resources, cultural norms, and technological advancements. As healthcare evolves towards patient-centered models, understanding the effects of healthcare communication strategies on patient caregiver satisfaction is increasingly important [3]. This review focuses on the communication practices healthcare professionals use to inform patient caregivers when the patient is taken to the operating theater. Practices may include in-person briefings, phone calls, text messages, or digital notifications via apps and specialized platforms [4,5]. Technology-driven approaches, such as digital platforms and automated SMS notifications, provide real-time updates but may lack the personal interaction that patient caregivers value [6,7,8]. Conversely, verbal updates, while more personal, may be less frequent or informative, potentially leading to dissatisfaction when patient caregivers are left without timely or comprehensive updates [9,10]. Healthcare communication in this context involves understanding and relaying the patient’s current location (e.g., surgical suites, operating rooms, operating theaters, and postoperative ICUs) and condition to patient caregivers. Patient caregiver satisfaction refers to the degree to which caregivers feel informed, reassured, and supported by the communication they receive [6,11]. The term patient caregiver in this context, also referred to as family caregiver or caregiver, is an individual, often a family member, such as spouse, parents, siblings, or friend, who accompanies patients to the surgery facility and provides emotional, informational, and logistical support, as well as care during their stay. 

Satisfaction is typically assessed through standardized instruments or surveys that evaluate dimensions such as timeliness, clarity, comprehensiveness, and the emotional impact of communication [7]. While existing research predominantly focuses on communication with patients, a significant gap remains in understanding its effects on caregivers [8,12]. While specific studies investigated caregiver anxiety and stress within critical care environments, there remains a scarcity of evidence regarding the impact of diverse communication practices on caregiver satisfaction across various healthcare settings globally [9,13,14]. The variation in communication methods employed within surgical settings highlights the importance of assessing the global trends in these practices and their influence on caregiver satisfaction [10,15].

In a preliminary search of MEDLINE, the Cochrane database of systematic reviews, and JBI evidence synthesis databases revealed no current or ongoing systematic or scoping reviews specifically on communication practices that meet the unique needs of patient caregivers [16]. Given the increasing adoption of digital communication tools in healthcare, evaluating their influence on patient caregiver satisfaction across different cultural and healthcare environments is crucial [17]. This scoping review aims to assess global communication practices in surgical waiting areas and their impact on patient caregiver satisfaction. Specifically, it will map the types of communication strategies used, the contexts in which they are applied (such as patient location updates), and their perceived effectiveness in enhancing patient caregiver satisfaction. The review highlights well-established practices and emerging trends, identifying areas where further research could improve patient caregiver communication. 

There are many existing reviews that primarily focus on patient outcomes or the stress experienced by patient caregivers in ICU and palliative care settings, where communication with patient caregivers is essential. However, there is limited evidence on communication strategies used in surgical waiting areas, where real-time updates are critical. This scoping review aims to map global communication practices used to inform patient caregivers during surgery and examine their impact on patient caregiver satisfaction.

A scoping review is appropriate due to the emerging nature of this research in patient caregiver-focused communication strategies, study settings, and the diversity of study designs, making it unsuitable for a systematic review. This approach allows comprehensive mapping of communication practices and identification of research gaps. Cultural norms significantly influence patient caregiver expectations and preferred communication styles. This review examines how these cultural differences affect patient caregiver differences, for instance, some cultures prioritize personal interactions and verbal updates, while others prefer digital updates more highly.

## 2. Methodology

### 2.1. Study Design

The methodology and stages for this scoping review were guided by the Joanna Briggs Institute (JBI)’s “methodology for scoping reviews” [18]. The final output adhered to the Preferred Reporting Items for Systematic Reviews and Meta-Analysis extension for Scoping Reviews (PRISMA-ScR) checklist [19]. Given the heterogeneity of evidence on patient caregiver communication, a scoping review is ideal for consolidating findings across diverse study designs and geographic settings. The review title has been registered in the OSF registry (osf.io/2f4t6, date registered: 22 November 2024). No protocol has been published for this research.

### 2.2. Review Question

1. What global communication practices have been adopted in surgical waiting areas of healthcare settings?
2. What is the impact of these communication practices on patient caregiver satisfaction?

### 2.3. Eligibility Criteria

Table 1 shows the eligibility criteria.

### 2.4. Search Strategy

An extensive search strategy was designed for this review, incorporating medical subject headings (MeSH) and carefully selected free-text keywords. To ensure the accuracy and breadth of the terminology, the search plan was critically reviewed by an experienced academic librarian specializing in health sciences. A systematic three-stage search process was implemented to optimize the retrieval of relevant studies. In the first stage, a range of major databases was queried using a comprehensive set of terms, including “Surgical Patient Caregivers”, “Communication Practices”, “Real-Time Updates”, “Patient Caregiver Satisfaction”, and “Surgical Care Settings”. Titles and abstracts were examined to identify key phrases, and associated indexing terms were analyzed to refine and broaden the search vocabulary. In the second stage, this expanded list of keywords and indexing terms was systematically employed across all selected databases to ensure thorough coverage. The final phase consisted of manually examining the reference lists of the selected articles and reports to uncover additional studies that might not have been retrieved through the electronic search. The databases consulted included PubMed, Scopus, Web of Science, Embase, the Cumulative Index to Nursing and Allied Health Literature (CINAHL), and ProQuest. These databases were chosen for their strong relevance to healthcare research and their ability to offer access to various sources, including peer-reviewed articles, interdisciplinary studies, and grey literature. The ProQuest database was used to identify the relevant grey literature, including theses and conference abstracts, on the current topic. This systematic approach was designed to enable a comprehensive and globally inclusive examination of communication practices within surgical and clinical healthcare settings.

### 2.5. Study Selection

Following the initial search, duplicate records were identified and excluded by cross-checking the author names, article titles, and publication periods. Two reviewers conducted the study selection independently, following a two-step screening process. In the first stage, the reviewers assessed the relevance of each study based on its title. Studies that appeared to meet the inclusion criteria then proceeded to the second stage, where the full abstracts were examined to determine their suitability for inclusion. Articles deemed eligible were further reviewed to ensure alignment with the predetermined criteria. Any discrepancies between reviewers regarding study eligibility were resolved through collaborative discussion until consensus was achieved. 

## 3. Results

### 3.1. Selection and Inclusion of Studies

**Figure 1:**
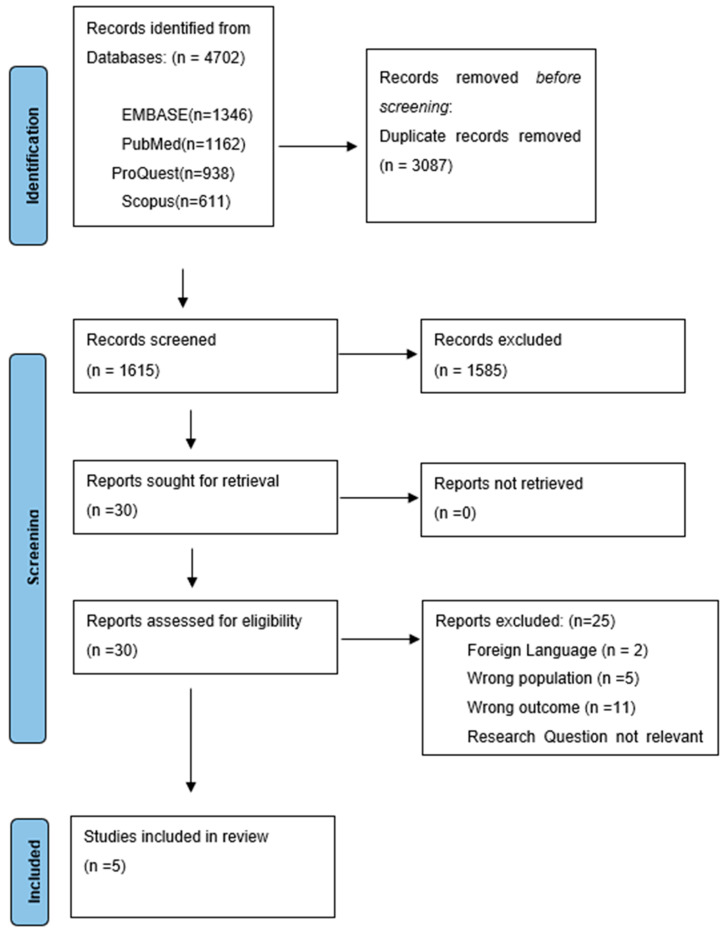
PRISMA Flow diagram.

A total of 4702 records were identified through comprehensive database searches, specifically from EMBASE (*n* = 1346), PubMed (*n* = 1162), ProQuest (*n* = 938), and Scopus (*n* = 611) (Supplementary Materials). After removing duplicates (*n* = 3087), 1615 unique records remained for title and abstract screening. During the title and abstract screening phase, 1585 records were excluded based on predefined eligibility criteria. Following this initial screening, 30 full-text reports were sought for retrieval. All targeted reports were successfully obtained and subjected to detailed full-text screening. Upon full-text assessment, 25 reports were excluded for various reasons: 2 were in foreign languages, 5 did not meet the specified population criteria, 11 had outcomes that were not relevant to the focus of this review, 4 posed research questions that were not applicable, and 3 were classified as background articles. Consequently, five studies met all eligibility criteria and were included in the final review, forming the basis of our analysis. The study’s inclusion and exclusion processes are depicted below in a flow diagram (Figure 1) based on PRISMA guidelines, ensuring transparency and reproducibility.

### 3.2. Synthesis of the Included Studies

A synthesis of five selected studies revealed diverse communication strategies to enhance the patient caregiver experience during surgical procedures. These interventions primarily target anxiety reduction and satisfaction improvement, as mentioned in Table 2. 

#### 3.2.1. Study Designs and Settings

The research settings varied, including operating theaters, emergency departments (ED), and surgical wards. The studies were conducted geographically across several countries, namely Iran, Canada, Switzerland, and the United States [20,21,22,23,24]. Among the five studies reviewed, three utilized cross-sectional or survey-based designs, one employed a randomized controlled trial (RCT), and another adopted a mixed-methods approach [22,23]. The study design varied among the included studies. The validation of the communication strategy was not available in the included studies. The cross-sectional study focused on the opinions rather than the communication methods, thus providing the outcome of the method rather than the method itself. 

#### 3.2.2. Traditional Versus Digital Communication Approaches 

In studies 1 (Canada) and 5 (Switzerland), digital communication through SMS-based intraoperative updates was utilized [20,24]. Study 2 examined conventional methods, such as in-person conversations and telephone updates, which were often unstructured and inconsistent [21]. Study 3 (Iran) incorporated both digital approaches (video-based education) and structured verbal updates provided periodically [22]. In study 4 (United States), significant communication gaps were observed during postoperative phases, characterized by informal interactions, such as brief hallway conversations and sporadic phone calls [23]. 

#### 3.2.3. Focus of Assessment and Measurement Tools

The primary focus of the included studies was the evaluation of caregiver anxiety and satisfaction. Anxiety was assessed in studies 1, 3, and 5 using validated instruments such as the State-Trait Anxiety Inventory (STAI) and a single-item Likert-type anxiety scale [20,22,24]. Caregiver satisfaction was a key outcome in studies 1, 2, and 5, typically measured through tailored survey instruments drawing from frameworks such as the unified theory of acceptance and use of technology (UTAUT) [20,21,24].

#### 3.2.4. Outcomes and Impact Measurement

A study conducted in Ethiopia reported a significant prevalence of preoperative anxiety among caregivers, with 74.2% of participants surpassing the clinical anxiety threshold (STAI score ≥ 44), indicating substantial concerns about their children’s anaesthesia and surgery. The research identified several key factors strongly correlated with elevated anxiety levels, including maternal status (adjusted odds ratio (AOR) = 4.45), lack of information about anaesthesia (AOR = 7.02), having a child under the age of one (AOR = 4.10), and the caregiver’s occupation as a farmer (AOR = 9.73). These findings emphasize the importance of healthcare providers recognizing various factors that influence caregiver anxiety when interacting with parents during the perioperative period. Thus, improving communication and offering tailored support can help alleviate caregiver stress, ultimately enhancing the surgical experience for both parents and children [20]. 

A study conducted in Canada on automated intraoperative short messaging service (SMS) updates revealed several key outcomes and impact measurements. Caregivers reported high satisfaction rates, with 70.8% finding the number of messages adequate, 74.4% considering them clear, and 71.6% feeling informed about their loved ones’ operations. The overall satisfaction score averaged 4.5 out of 5, indicating a strong positive reception of the SMS communication system. Additionally, caregivers experienced an anxiety reduction, scoring an average of 8.2 out of 10 regarding the effectiveness of the SMS messages in alleviating their concerns during surgery. Engagement with the SMS system was also notable, as caregivers for 6149 (75.6%) out of 8129 scheduled surgeries opted to receive updates, suggesting that the service was well-received. Thirty-four thousand one hundred twenty-nine messages were sent, with only 3.3% of caregivers reporting technical errors, indicating a generally reliable system. Feedback was collected through a satisfaction survey sent to caregivers one business day after surgery, achieving a response rate of 34% (2088 out of 6149 caregivers), which, while lower than average, still provided valuable insights. The study suggests future research should include a randomized prospective study to assess further the impact of the SMS initiative on caregiver anxiety and satisfaction, highlighting a commitment to continuous improvement in healthcare communication. The successful implementation of an automated SMS messaging system alleviated caregiver anxiety. Additionally, it significantly enhanced satisfaction with surgical care, demonstrating the transformative potential of digital communication in healthcare settings [21].

A study in Iran aimed to evaluate the effectiveness of video training and intraoperative progress reports on reducing anxiety among family caregivers waiting for relatives undergoing surgery. The outcomes were measured using the Spielberger State-Trait Anxiety Inventory (STAI), which assessed both state and trait anxiety levels. Results indicate a statistically significant decrease in state anxiety for both the video training group (*p* < 0.001) and the intraoperative progress report group (*p* < 0.001) after the interventions compared to their anxiety levels before the interventions. However, there was no significant difference in state and trait anxiety levels among the groups after the interventions (*p* > 0.05). The study included 102 eligible participants, who were randomly allocated to three groups: The study featured a three-armed design consisting of the video training group, which received educational videos about the surgical process and postoperative care; the intraoperative progress report group, which obtained updates on their relatives’ condition and surgical progress during the procedure; and the control group, which received no information unless specifically requested, with all participants’ data included in the final analysis. The findings suggest that both interventions are effective in alleviating state anxiety, highlighting the importance of providing information and support to family caregivers during the surgical process [22].

A study conducted in the United States aimed to evaluate and enhance perioperative communication practices between surgeons and caregivers at a single-site academic medical center. The findings indicate that telephone contact was the predominant method of postoperative communication, employed by 66.2% of surgeons, whereas in-person interactions were notably less frequent. Specifically, only 28.6% of uninsured patients received face-to-face communication compared to 34.6% of insured patients (*p* < 0.0001). Significant variations were also identified based on the nature of the surgical procedure; in-person discussions occurred in 34.4% of elective surgeries, compared to 31.9% during emergency surgeries (*p* = 0.006). Most communications with caregivers happened in the waiting area, with 81.0% of telephone updates delivered there, in contrast to only 19.0% of in-person consultations within designated consult rooms. Nominal group sessions revealed strong correlations between communication themes identified by surgeons and caregivers (r = 0.86) and between staff and caregivers (r = 0.84). The results highlight the pressing need for improved perioperative communication strategies to meet caregiver expectations better and to improve overall satisfaction. The study findings reveal that communication approaches varied notably by demographic groups and specialities, thus highlighting the need for standardized protocols to ensure equitable patient-centered care [23].

A study in Switzerland sought to assess the impact of a semi-automated text messaging system on improving patient satisfaction regarding wait times in the emergency department. Data were collected from 110 valid responses, comprising 100 patients and 10 caregivers, revealing a notably high patient satisfaction rate of 97%. Among the participants, 80% reported waiting within the hospital premises, 13% waited outside the facility, and 2% opted to wait at home. Regarding perceived wait times, 49% of respondents indicated that the actual wait was longer than expected, while 25% reported that the actual wait corresponded to the announced time. In total, 72% of participants affirmed a clear understanding of the SMS content, underscoring its effectiveness in facilitating communication. Analysis using the unified theory of acceptance and use of technology (UTAUT) model revealed an average performance expectancy score of 3.6 and an effort expectancy score of 6.0, suggesting that although the system was considered user-friendly, concerns regarding increased caregiver workload impeded its broader adoption. Overall, the findings indicate that the SMS system holds considerable promise for enhancing the patient waiting experience; however, additional measures are necessary to address caregiver workload challenges associated with its use [24].

Evidence across diverse global settings suggests that timely and structured communication interventions during surgical or emergency procedures consistently improve patient caregiver satisfaction and reduce anxiety, though modes and outcomes vary. For instance, in Ethiopian and Iranian studies, patient caregiver anxiety emerged as a critical concern, where lack of anaesthesia information (AOR = 7.02) and patient caregiver role, such as maternal status (AOR = 4.45), correlated strongly with elevated anxiety levels [20,22]. Digital interventions such as intraoperative SMS updates in Canada showed higher satisfaction scores (mean score 4.5/5) and anxiety reduction (mean 8.2/10), while in Iran, video training and real-time updates significantly lowered state anxiety (*p* < 0.001) [21,22]. Contrastingly, in the U.S., reliance on phone calls (66.2%) over in-person interactions revealed disparities, and also the uninsured patient caregivers and those involved in emergency surgeries received fewer in-person updates (*p* < 0.0001 and *p* = 0.006), reflecting inequities in communication practices [23]. While the patient caregivers in Switzerland appreciated SMS updates (72% found them clear), concerns over patient caregiver workload impeded broader implementation [24]. The above findings highlight the need for structured communication approaches, especially digital communication interventions, in enhancing the patient caregiver experiences, while also emphasizing the importance of equitable, culturally sensitive strategies adaptable to various healthcare settings.

## 4. Discussion

This scoping review highlights significant diversity in the communication strategies employed within surgical settings worldwide. Approaches to communication varied from unstructured verbal updates and telephone calls to more formalized digital solutions, including real-time SMS notifications and video-based educational tools. These differences can be attributed to variations in healthcare infrastructure, access to technological resources, and cultural norms regarding caregiver participation in clinical care [5,25]. These contextual factors emphasize the need for communication strategies to be contextually adapted, ensuring they align with the specific needs and expectations of patient caregivers in each healthcare environment. A consistent finding across the included studies was the impact of communication quality on caregiver satisfaction and anxiety levels. Communication that was clear, timely, and structured contributed significantly to reducing uncertainty and emotional distress among caregivers waiting for surgical updates [26,27]. Specifically, studies incorporating digital interventions, such as SMS-based real-time updates, reported notable improvements in patient caregiver satisfaction and measurable reductions in anxiety, thereby underscoring the transformative potential of well-designed communication strategies in enhancing the caregiver experience [28]. 

The effectiveness of digital communication tools was particularly evident in studies employing SMS notifications and video-based educational interventions. These technologies offered real-time and consistent updates. They were generally well-received by caregivers and regarded as effective in bridging communication gaps during surgical procedures [21,28,29]. These findings are consistent with an expanding body of literature emphasizing the benefits of digital health technologies in enhancing transparency and ensuring continuity of care. This is especially true in high-stress environments such as surgical waiting areas. Nonetheless, the review also identified notable gaps in communication during critical perioperative phases, especially the postoperative period. Patient caregivers frequently reported reliance on informal conversations or experienced delays in receiving updates, resulting in confusion and dissatisfaction [23]. These lapses reflect broader communication challenges observed across healthcare systems. They highlight the need for the implementation of standardized protocols throughout the entire surgical journey [30]. 

While digital interventions show considerable promise, several barriers to effective implementation persist. These include concerns related to user-friendliness, varying levels of digital literacy among caregivers, and increased operational burdens on clinical staff. Without a user-centered design and sufficient infrastructural and human resource support, such technological innovations risk becoming counterproductive [31,32,33]. Therefore, technology-based solutions must be accessible and integrated seamlessly into existing workflows. A further limitation identified within the literature pertains to the inconsistent measurement of caregiver satisfaction. The use of diverse assessment tools, such as Likert scales and customized questionnaires, led to challenges in cross-study comparison. This highlights the urgent need to develop standardized, culturally adaptable, and validated instruments to measure caregiver satisfaction reliably across different healthcare systems. 

Cultural and socioeconomic factors also influenced caregivers’ perceptions of communication quality significantly. For instance, patient caregivers with limited formal education or from rural backgrounds reported higher levels of anxiety. This was especially true in situations where communication was infrequent or lacked personalization [20]. These findings reinforce the importance of tailoring communication strategies to align with the patient caregivers’ linguistic, cultural, and information needs [33]. Emerging evidence suggests that multimodal communication strategies that combine conventional methods, such as face-to-face verbal updates, with digital tools such as SMS alerts and instructional videos, offer the most balanced approach. This hybrid model helps maintain personal interaction while also improving timeliness and operational efficiency [21,22,34]. 

Notably, the review reveals a substantial gap in research from low and middle-income countries, where caregiver perspectives and communication challenges remain underrepresented. Existing studies are often limited in scope, with few examining long-term outcomes or post-discharge satisfaction. Future investigations should focus on longitudinal and randomized controlled studies conducted in diverse healthcare settings to generate robust, generalizable evidence for best practices. These findings carry important implications for both policy and clinical practice. Healthcare institutions, particularly surgical departments, are encouraged to institutionalize communication protocols involving caregivers throughout the surgical process. Measures such as implementing real-time update systems, establishing structured feedback mechanisms, and providing training to healthcare professionals in effective communication techniques may contribute significantly to improving caregiver satisfaction and the overall quality of surgical care [35,36]. The study findings highlight the fact that the communication methods not only varied in different forms, such as verbal updates to digital communication platforms, but also differed in their effectiveness across various healthcare settings. It was observed that the patient caregivers in rural or low-resource areas faced challenges such as limited digital literacy skills, whereas the implementation of digital communication tools, such as SMS and video updates, was more successful among patient caregivers in the urban and well-resourced areas. Even factors such as cultural contexts, cultural norms, and patient caregiver education levels defined how communication was perceived. Hence, there is a need to have tailored communication strategies that are more specific to the social and technological contexts to ensure more equitable and effective patient caregiver involvement. Recent advances in digital technology transformed the field of surgery. FDA-approved AI systems for endoscopy and wearable biosensors in clinical trials illustrated the potential of these breakthroughs. Specialties such as gastroenterology and radiology are already embracing innovations such as machine learning, computer vision, wearable devices, remote patient monitoring, and virtual and augmented reality in surgery [37]. The healthcare industry is transitioning from hospital-centered to patient-centric models, largely driven by the Internet of Things (IoT). IoT-enabled systems, equipped with sensors and devices, have proven to provide continuous 24 h monitoring, improving patient care, especially in long-term settings [38]. Machine learning (ML) models have been shown to improve surgical outcomes, with AI aiding diagnosis and real-time intraoperative analysis in cardiology and surgery [37,39]. Preoperatively, ML helps to predict risks and reduce presurgical costs via telemedicine, whereas postoperatively, ML has been shown to improve ICU outcomes by predicting sepsis and readmissions, while AI and IoT technologies helped, particularly during COVID-19, enhance patient classification and remote monitoring [40,41]. The implementation of digital technologies, such as the Wearable IoT Care System, has not only helped clinicians, surgeons, and healthcare providers, but also helps to monitor the vital signs of the patients and supports real-time communication between elderly patients and patient caregivers [42]. The IoT plays a crucial role in remote pregnancy care, using wearable sensors for continuous monitoring of maternal and fetal health, provide medication reminders, prenatal and postnatal care, track vital signs and medication management, and provide real-time data transmission to healthcare providers, reducing the need for frequent hospital visits [42,43]. Although the use of radio frequency identification (RFID) is gaining traction in hospitals for data transfer and patient identification, it also enhances efficiency and service quality, but poses challenges related to high costs and complexity. Other factors, such as technological readiness, organizational needs, and environmental factors, also have crucial influences on RFID adoption in healthcare settings. Research has shown that larger hospitals with resources are more likely to adopt RFID, being influenced by government policies and market competition [44]. Thus, for the successful implementation of digital communication tools in healthcare settings, the inclusion of user-friendly interfaces and systems is essential to provide more transparent and collaborative care to the patients as well as the patient caregivers. In low-resource areas and multilingual healthcare settings, it is essential to understand the digital health literacy levels, cultural norms and expectations, and local and regional languages of the patient caregiver populations in order to design more specific communication strategies that are adaptable and accessible to such healthcare settings.

### Limitations

There are a few limitations in this scoping review that should be considered. There are a limited number of studies included in this review (n = 5), which restricts the evidence base and also constrains the generalizability of the findings across various healthcare settings and study populations. Furthermore, the ability to draw definitive conclusions from the efficacy of various communication interventions was restricted due to the methodological heterogeneity among the selected studies. Since only the studies that were published in the last 10 years were included, potentially excluding earlier foundational research, which could offer valuable insights into the evolution of different communication methods in healthcare environments. Additionally, the studies that were related to electronic health records (EHR) and telemedicine were excluded, thus the applicability of the study findings only to the existing and emerging technological innovations. Another significant limitation is the absence of a formal quality assessment of the included studies, which might undermine the strength and validity of the study findings. Further research in the form of a systematic review is recommended to evaluate the individual communication interventions for assessing their effectiveness and impact on the patient caregivers, with the aim of identifying approaches that lead to improved outcomes. This review lacked the absence of the quality appraisal of the studies and the limited generalizability due to the smaller number of studies.

## 5. Conclusions

During stressful perioperative periods, effective communication practices between surgical staff and patient caregivers in surgical care environments are essential. This scoping review highlights that when tailored to the cultural and contextual communication needs of the patient caregivers, both the traditional and digital communication approaches can have a significant impact on their satisfaction levels. Despite technological advancements in communication practices, the importance of having personalized and culturally sensitive information pertaining to the needs of the patient caregivers, where they feel emotionally supported, cared for, and well-informed about their patient’s updates, plays a vital role in enhancing their experience in surgical care settings. There should be future studies that focus on having standardized communication assessment tools, investigating long-term outcomes, and bridging the communication disparities and inequities, especially in middle and low-income resource countries. Implementing culturally sensitive multimodal communication strategies can contribute to enhancing the patient caregiver experience and better patient care and outcomes. Future implications should include having tailored and standardized communication frameworks that include clear and consistent communication practices across all healthcare settings globally, and thus ensuring digitally accessible and equitable distribution of information delivery among the underserved patient caregiver population groups worldwide, thus bridging the communication gaps in the perioperative period and enhancing the overall patient caregiver experiences.

## Figures and Tables

**Table 1:** Eligibility criteria.

Framework	Inclusion Criteria	Exclusion Criteria
**Population**	Studies focusing on communication practices between healthcare staff and patient caregivers.	Studies focusing on communication between healthcare staff and studies involving only patients, excluding patient caregivers, will not be considered.
**Concept**	Studies that report satisfaction levels in patient caregivers, even if they report anxiety, should also consider satisfaction as one of the outcome measures. Studies focusing on various types of healthcare communication practices used in clinical healthcare settings such as verbal, non-verbal, written, electronic and digital communication methods such as text messaging (SMS), secure messaging platforms such as HIPAA-compliant messaging services, automated communication systems such as audio-visual boards, mobile health (mHealth) apps and wearable devices, and remote monitoring tools such as RFID technology will be included. Studies involving healthcare staff directly involved in patient care, clinical staff, support staff, and surgical staff will be included.	Studies that report only anxiety or other patient-related outcomes but do not report satisfaction levels in patient caregivers. Studies focusing on telemedicine and electronic health records (EHRs) will not be considered. Studies involving healthcare staff not directly involved in patient care will be excluded from this review.
**Context**	Studies reporting communication practices from different global clinical healthcare settings and cultural contexts will be included to understand their impact on patient caregiver satisfaction.	Studies reporting communication practices occurring in the non-clinical areas of healthcare settings will not be included.
**Type of Studies**	Last 10 years of studies—identifying trends, patterns, and shifts more clearly, significant advancements or technological changes, studies published in the English language, qualitative and quantitative studies, studies with both experimental and quasi-experimental study designs, including randomized controlled trials, observational studies including prospective and retrospective cohort studies, case-control studies, and analytical cross-sectional studies; descriptive observational study designs include case series, individual case reports, and descriptive cross-sectional studies, grey literature, book chapters, a thesis, references from the review papers, and dissertations will all be included.	Review papers, primary studies, systematic reviews, narrative reviews and other review studies, as well as documentaries and case studies, will not be included.

**Table 2:** Characteristics of included studies.

Sl No	Author/Year of Publication	Place/ Country	Study Design and Sample Size	Study Settings	Communication Practices (Traditional/Digital)	Patient Caregiver (PC) Anxiety/Satisfaction	Outcome Measures
1	Nigussie Simeneh Endalew (2020) [20]	Ethiopia	Cross-sectional study design Sample size—194	Department of Anaesthesia	Traditional	PC anxiety—state and trait anxiety inventory (STAI)	The overall prevalence of clinical-level anxiety was found to be 74.2%, with an STAI-State score of 44 and above indicating high anxiety levels.
2	Éric Tchouaket Nguemeleu (2022) [21]	Canada	Quality improvement initiative Sample size—884	Surgical Department	Digital—short messaging service (SMS)	PC anxiety— PC satisfaction	Post-intervention scores indicated a high satisfaction score of 4.5 out of 5 and an anxiety reduction score of 8.2 out of 10. The overall satisfaction score stated a 90% satisfaction rate.
3	Maryam Maleki (2022) [22]	Iran	Randomized controlled trial Sample size—102	Surgical Department	Traditional—verbal communication and digital—video training	PC anxiety—Spielberger State-Trait Anxiety inventory (STAI)	Pre-intervention, the video training group showed a statistically significant decrease in state anxiety (*p* < 0.001). No significant difference in anxiety scores across groups after the intervention (*p* > 0.05), indicating the interventions effectively reduced state anxiety but did not significantly differ among groups post-intervention.
4	Nisha Patel (2017) [23]	United States	Mixed-method study Sample size— PC—35, surgeons—13, and waiting room personnel—9	An academic medical center	Traditional—in-person conversations and digital—phone calls	PC satisfaction	Phone call contact was the most common method for surgeons, utilized in 66.2% of cases compared to other communication methods. Need for a revised perioperative communication process tailored to both surgeons and patient caregivers.
5	Jessica Rochat (2021) [24]	Switzerland	Cross-sectional, descriptive study Sample size— Patients—100 PC—10	Adult emergency department	Digital—semi-automatic text message (SMS) system	PC satisfaction—Unified Theory of Acceptance and Use of Technology (UTAUT) questionnaire	Patient satisfaction levels: 97% of patients were satisfied with the SMS system. Patient caregiver satisfaction levels: average score of 6.0 (scale of 1 to 7), indicating that caregivers found the system easy to use.

## Data Availability

Not applicable.

## Supplementary Materials

The following supporting information can be downloaded at: https://www.mdpi.com/article/10.3390/healthcare13121408/s1.
