# Supplementary material for: Global Communication Practices and Their Impact on Patient Caregivers’ Satisfaction in the Surgical Waiting Area: A Scoping Review
Source: Healthcare (Basel). 2025 Jun 12;13(12):1408. doi: 10.3390/healthcare13121408 (PMC12193462; doi:10.3390/healthcare13121408)
Supplement: Supplementary file 1 [file healthcare-13-01408-s001.zip › healthcare-3658477-supplementary.pdf]

Supplementary Materials

Database	Search	Hits
EMBASE	('waited':ti,ab,kw,de,dn,df,mn,tn OR 'waiting':ti,ab,kw,de,dn,df,mn,tn OR 'waits':ti,ab,kw,de,dn,df,mn,tn OR 'hospital admission'/exp OR 'wait list':ti,ab,kw,de,dn,df,mn,tn) AND ('caregiver'/exp OR 'caregivers':ti,ab,kw,de,dn,df,mn,tn OR 'caregiver':ti,ab,kw,de,dn,df,mn,tn OR 'caregiving':ti,ab,kw,de,dn,df,mn,tn) AND ('surgery' OR 'surgery':ti,ab,kw OR 'surgery'/exp OR 'general surgery'/exp OR 'surg*':ti,ab,kw)	923
PubMed	("waited"[Text Word] OR "waiting"[Text Word] OR "waits"[Text Word] OR "waiting lists"[MeSH Terms] OR "wait list"[Text Word]) AND ("caregivers"[MeSH Terms] OR "caregivers"[Text Word] OR "caregiver"[Text Word] OR "caregiving"[Text Word]) AND ("surgery"[MeSH Subheading] OR "surgery"[Title/Abstract] OR "surgical procedures, operative"[MeSH Terms] OR "general surgery"[MeSH Terms] OR "surg*"[Title/Abstract])	138
WoS	(ALL=waited OR ALL=waiting OR ALL=waits OR ALL="waiting lists" OR ALL="wait list") AND (ALL=caregivers OR ALL=caregivers OR ALL=caregiver OR ALL=caregiving) AND (ALL=surgery OR (TI=surgery OR AB=surgery) OR ALL="surgical procedures, operative" OR ALL="general surgery" OR (TI=surg* OR AB=surg*))	122
Scopus	(TITLE-ABS-KEY(waited) OR TITLE-ABS-KEY(waiting) OR TITLE-ABS-KEY(waits) OR INDEXTERMS("waiting lists") OR TITLE-ABS-KEY("wait list")) AND (INDEXTERMS(caregivers) OR TITLE-ABS-KEY(caregivers) OR TITLE-ABS-KEY(caregiver) OR TITLE-ABS-KEY(caregiving)) AND (INDEXTERMS(surgery) OR TITLE-ABS(surgery) OR INDEXTERMS("surgical procedures, operative") OR INDEXTERMS("general surgery") OR TITLE-ABS(surg*))	111
CINAHL	(waited OR waiting OR waits OR (MH "waiting lists+") OR "wait list") AND ((MH caregivers+) OR caregivers OR caregiver OR caregiving) AND ((MW surgery) OR (TI surgery OR AB surgery) OR (MH "surgical procedures, operative+") OR (MH "general surgery+") OR (TI surg* OR AB surg*))	119
ProQuest	("waited"[Text Word] OR "waiting"[Text Word] OR "waits"[Text Word] OR "waiting lists"[MeSH Terms] OR "wait list"[Text Word]) AND ("caregivers"[MeSH Terms] OR "caregivers"[Text Word] OR "caregiver"[Text Word] OR "caregiving"[Text Word]) AND ("surgery"[MeSH Subheading] OR "surgery"[Title/Abstract] OR "surgical procedures, operative"[MeSH Terms] OR "general surgery"[MeSH Terms] OR "surg*"[Title/Abstract])	938
